# In natural interaction with embodied robots, we prefer it when they follow our gaze: a gaze-contingent mobile eyetracking study

**DOI:** 10.1098/rstb.2018.0036

**Published:** 2019-03-11

**Authors:** Cesco Willemse, Agnieszka Wykowska

**Affiliations:** Social Cognition in Human-Robot Interaction, Istituto Italiano di Tecnologia, Via Enrico Melen 83, 16040 Genova, Italy

**Keywords:** joint attention, gaze leading, mobile eyetracking, social robots, gaze contingency

## Abstract

Initiating joint attention by leading someone's gaze is a rewarding experience which facilitates social interaction. Here, we investigate this experience of leading an agent's gaze while applying a more realistic paradigm than traditional screen-based experiments. We used an embodied robot as our main stimulus and recorded participants' eye movements. Participants sat opposite a robot that had either of two ‘identities’—‘Jimmy’ or ‘Dylan’. Participants were asked to look at either of two objects presented on screens to the left and the right of the robot. Jimmy then looked at the same object in 80% of the trials and at the other object in the remaining 20%. For Dylan, this proportion was reversed. Upon fixating on the object of choice, participants were asked to look back at the robot's face. We found that return-to-face saccades were conducted earlier towards Jimmy *when* he followed the gaze compared with when he did not. For Dylan, there was no such effect. Additional measures indicated that our participants also preferred Jimmy and liked him better. This study demonstrates (a) the potential of technological advances to examine joint attention where ecological validity meets experimental control, and (b) that social reorienting is enhanced when we initiate joint attention.

This article is part of the theme issue ‘From social brains to social robots: applying neurocognitive insights to human–robot interaction’.

## 1. Introduction

Humans are efficient in processing social information from others' gaze [1,2]. The gaze-cueing effect, in which spatial orienting is facilitated by receiving the redirection of an agent's gaze [3,4] is one example. But, shifts of gaze-direction can also be *signalled* to an agent in a bid to establish joint attention. In typical gaze-leading paradigms, participants redirect their gaze towards a stimulus in the peripheral view, after which an avatar on the screen looks at either the same object or a different one [5,6]. Successful initiation of joint attention in this manner has been found to increase brain activation related to hedonistic reward [6] and to increase subjective experiences of liking and preference of the responding avatar [5,7,8].

Moreover, people frequently took less time to re-orient their gaze back from the object towards the avatar when this agent followed the gaze, compared with when it did not [5,7]. This effect is also modulated by the typically experienced response of the avatar: the average onset latency of these return-to-face saccades is shorter towards avatars that usually follow the participant's gaze towards an object than to those who typically looked at the other object [8].

Whereas past gaze-leading studies had their stimuli presented on a computer screen with the use of a desktop-mounted eyetracker, our current paper takes advantage of two technical advances to mimic realistic scenarios more closely. Firstly, we used a humanoid robot as an embodied agent, introducing social presence, to examine ecologically valid aspects of social cognition [9,10]. Secondly, we used mobile eyetracking technology to allow a set-up that would be more ecologically valid and that would not rely on using a (single) screen, as that may not be fully representative of natural gaze allocation and attention in environments outside of the laboratory [11].

Another clear advantage of using embodied agents as stimuli, besides studying whether screen-based findings are analogous to human social cognition in more realistic scenarios, is the potential to examine whether human–robot interaction is analogous to human–human interaction. That is to say, examining this possible analogy here will give us a better understanding of the conditions under which robots evoke mechanisms of social cognition in the humans, whether robots are perceived as animate agents, or when they are anthropomorphized.

### (a) Goals of the current study

Technological advances in eyetracking afford a welcome invitation to study joint attention, established by gaze-leading, with a humanoid robot. This poses a number of questions and challenges, such as whether speeded return-to-face saccades for joint attention episodes and the associated increase in preference and likeability replicate in set-ups away from the screen. Another such question is whether robots are perceived as more human-like and in possession of mental states when they respond congruently to gaze-leading. Finally, a challenge lies in establishing whether a gaze-contingent experimental paradigm can be feasibly conducted with mobile eyetrackers. Specifically, the study presented here aims to shed light on the following questions:

*Do past screen-based findings replicate in a more naturalistic scenario with an embodied agent?* Speeded return-to-face saccades are a sensitive marker of attentional engagement with the agent. We previously also found that besides the gaze being followed on an *ad hoc* basis, people are also sensitive to whether the agent *usually* follows their gaze or not [8]. However, on the side-lines of the current reproducibility debate in psychology (e.g. [12–14]) in which one-one-one replication is encouraged, even less is currently known about whether previous findings in social cognition replicate in more naturalistic adaptations. One might argue that they would, as traditional experiments are often well-controlled and contain few confounding variables. However, they may not necessarily replicate. For example, it has been found that people look more at a person on a screen compared with the same person when he/she is present and can thus be actually interacted with [15]. This begs an invitation to explore past findings in gaze-leading with added social presence. Therefore, we designed an experiment with a more naturalistic social interaction scenario, where participants were seated in front of an *embodied* robot of human-like size.

The two robot ‘identities’ were manipulated as within-subjects factor: one identity who usually followed the gaze and one who usually did not. We expected our participants to be sensitive to these identities as well as the *ad hoc* contingency, in line with previous findings. Note, however, that, as mentioned above, replication of previous screen-based findings would not only be a ‘mere’ replication, but it would show that findings obtained in controlled (but artificial) set-ups generalize to more ecologically valid scenarios, with embodied agents that can manipulate the environment (in contrast to stimuli only presented on the screen). Moreover, it would serve as a proof of concept that traditional paradigms of cognitive experimental psychology can be successfully implemented in a human–robot-interaction scenario, with replicable results and adequate scientific rigour—a task that is not trivial, given the technological challenges of integrating various components of the set-up (the humanoid robot, an eyetracker, stimulus presentation and response collection software) with excellent temporal synchronization that is required.

*Does establishing joint attention with gaze-leading influence subjectively reported likeability or preference towards other agents?* It has been reported that successful gaze-leading affects subjective attributions towards an agent, such as likeability and preference, relative to non-following agents [5,7,8,16]. It could also be argued that people will anthropomorphize a robot more after a higher degree of joint attention [8]. Therefore, we subjected participants to questionnaire items assessing anthropomorphizing, likeability and preference. We expected to find a positive relationship between the amount of successfully initiating joint attention and subjective ratings of likeability, human-likeness, and preference between the two agents.

Additionally, we explored whether establishing joint attention with the robot increased the adoption of the intentional stance. Dennett [17] proposed the idea that humans adopt various stances or strategies when predicting or explaining observed behaviours of other systems. Various strategies are best suited to various systems. For explaining/predicting behaviour of other humans, the intentional strategy, which refers to mental states, works best. It might therefore be that humans also adopt the intentional stance towards humanoid robots, and that a certain type of social interaction with them might influence the likelihood of adopting the intentional stance. To examine this, we presented a theory of mind test and a recently developed intentional stance recently developed questionnaire [18] directly after the participants completed the experimental session with each robot identity.

## 2. Method

### (a) Participants

We set a sample size of *N* = 32 before commencing this study, based on our previous study [8]. We estimated that we would suffer approximately 10–20% data loss, even though this was difficult to predict, and therefore deliberately overshot recruitment, maximizing availability of the laboratory. In total, 37 participants (21 females, mean age (*M*_age_) = 24.4 years, s.d. = 3.89) took part in the study for a payment of €15. This study was conducted in accordance with the ethical approval from the local ethical committee (Comitato Etico Regione Liguria) and participants provided written informed consent to participate.

### (b) Materials

**Figure 1.**
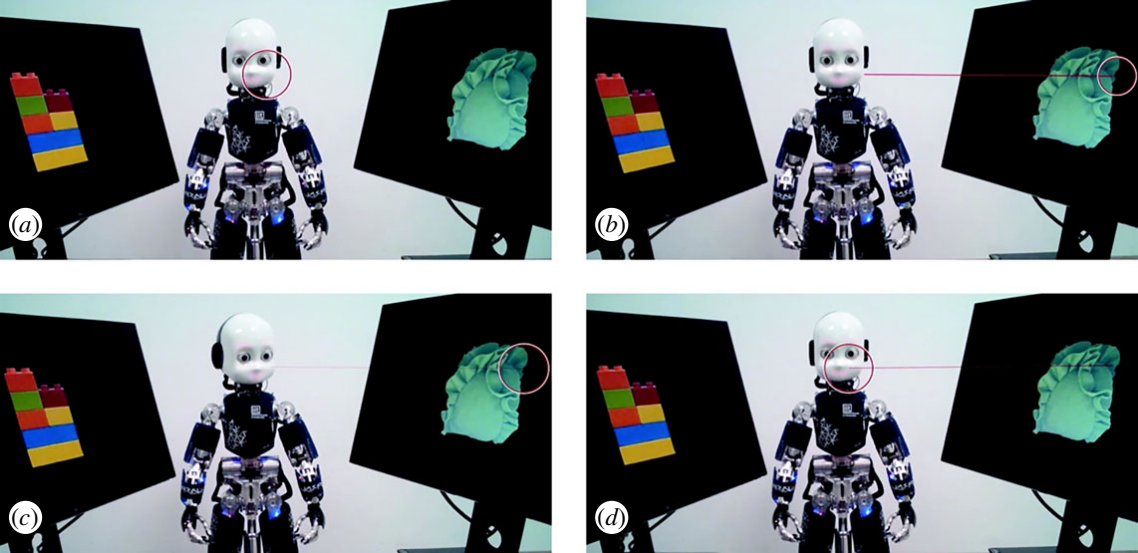
Trial sequence. Starting top-left: (*a*) The participants looked at the robot until they heard a beep. (*b*) They looked to the left or right object as quickly as possible. (*c*) iCub looked at an object (gaze-following example provided). (*d*) In their own time, the participants looked back at the robot's face (return-to-face saccade onset-time), upon which the robot looked at the participant again.

Participants sat opposite the iCub robot [19] at a distance of approximately 125 cm in a lit room, with a table in-between the robot and the participant. The iCub was mounted at a height such that his eyes were 122 cm from the floor, which we estimated to be roughly at eye level with most participants. Object images (720 pixels height, variable width, average 513 pixels, except for one object which was 720 pixels wide and 318 pixels high) were presented on two screens (27 inch, 2560 × 1440 pixels resolution, 144 Hz refresh rate). These screens were positioned one on each side of the table, so that the iCub and the participant were in-between the screens (see figure 1 for the set-up from the participant's perspective). The screens were tilted back by 15° from the vertical position, rotated 75° laterally and positioned 42 cm apart measured between the closest corners.

We used a pair of Tobii Pro Glasses 2 to record gaze data at a sampling rate of 100 Hz. Additionally, these data were streamed live to iCub via a Python controller (https://github.com/ddetommaso/TobiiProGlasses2_PyCtrl). Specifically, we divided the front-mounted camera image into three zones (left, centre and right; 30%, 40% and 30%, respectively). This information was sent to the presentation software (OpenSesame v.3.2; [20]) to control where the robot looked.

#### (i) Robot-related questionnaires and individual differences

A series of questionnaires was presented throughout the experiment. Firstly, we used the ‘InStance’ questionnaire devised to assess whether people adopt intentional stance towards the iCub robot [17]. This questionnaire presents 34 three-image sequences of iCub interacting with objects and/or people, and participants are asked to give an explanation for the displayed behaviour by sliding a slider on a rating scale either to a provided answer that has a mentalistic description, or to one that is mechanistic.

Additionally, we adapted the Yoni test of cognitive and affective theory of mind [21] and replaced the central original character with an iCub drawing to measure theory of mind towards the iCub. The Yoni test comprises 98 trials with first-order and second-order scenarios that assess both cognitive and affective theory of mind.

Furthermore, we used the likeability and anthropomorphism subscales of the Godspeed questionnaire [22]. These subscales comprise five items each with 1–5 Likert scale scoring between two antonymous adjectives (e.g. unpleasant—pleasant), so both subscales had a possible range of 5–25 with higher scores reflecting the attribution of more positive traits.

Finally, we assessed robot-preference with additional questions (which robot they preferred and why, which one they would think was meant to be the mentalistic/mechanistic one, and which one they would think was faster than the other). Other items served as a manipulation check, namely to assess whether participants noted the difference between conditions, whether they were aware of the nature of the experiment and whether they had participated in studies with the iCub before. All questionnaires were presented in Italian.

### (c) Procedure

After receiving the task instructions, participants were calibrated on the eyetracker, and a practice session started. The iCub looked up from a more downward position to mimic mutual gaze and participants were asked to look at the iCub's face, even when objects appeared on the screens, until they heard a beep (750 Hz, 100 ms, random onset between 750 ms and 1250 ms after stimulus onset). This beep acted as their cue to look at either the left or the right object as quickly as they could, using their eyes, but not their head, as much as possible. Looking direction was free choice, but they were asked not to look constantly towards the same direction. Live gaze samples were transmitted from the eyetracker to the experimental software via ethernet, and as soon as 10 samples in either the left or right zone were collected, iCub also turned his head—according to the trial condition—20° horizontally and 5° vertically (relative to the robot's frame of reference, mean movement onset-time was 127 ms) to give the impression that he was looking at one of the objects. Participants could look at the object for as long as they wanted, and were asked to then look back at the robot's face in their own time. The robot then turned his head forward again, after which participants pressed the spacebar to initiate the next trial. See figure 1 for a trial example, and the video demonstration available at https://osf.io/zxkwn and at https://youtu.be/rRZ9KdYnCes).

Whereas, in the practice session, the robot was anonymous (and introduced as such) and followed or unfollowed the participants’ gaze at random (50/50), participants did the task with a robot presented to them as either ‘Jimmy’ or ‘Dylan’ in the experimental session. The identity introduced as Jimmy followed the participants’ gaze to the same object in 80% of the trials, and Dylan in 20% of the trials, otherwise, they looked at the other object. Thus, Jimmy was an identity with whom joint attention was more often established than with Dylan.

The entire procedure was as follows. Participants did 16 practice trials, after which they started either with Jimmy or with Dylan (order counterbalanced between participants, two blocks of 40 trials for each). At the beginning of each block, the robot introduced himself (‘Hi, I am Jimmy/Dylan’). After two blocks, participants were taken to a computer to complete 17 items of the InStance questionnaire, which were randomly selected for each participant, the whole Yoni test and Godspeed items in that order. Each task was presented in such a way that it specifically referred to the robot with whom the participant had just done the task. In the meantime, the experimenter sat in the experimental room to ‘change the robot identity’ and kept up appearances by typing vigorously.^1^ Next, the participant did the task with the other identity and then filled out the questionnaires about the other identity. Finally, he/she completed the additional preference questions/manipulation checks, and was debriefed. Participants wore the eyetracking glasses for 10–15 min per identity. Altogether, the experimental session took approximately 90 min.

### (d) Data processing

**Figure 2.**
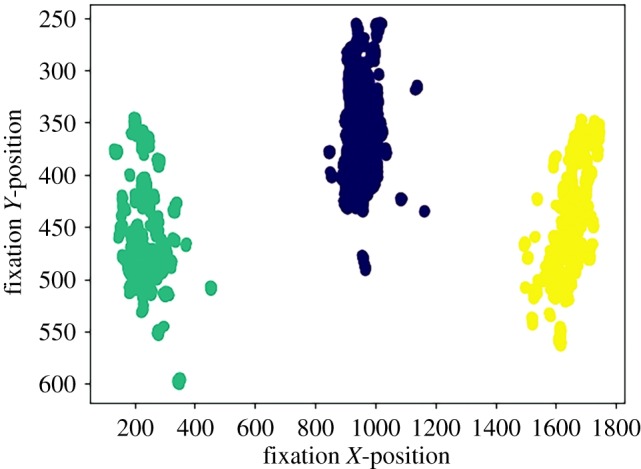
Example output of a *K*-means cluster classification of one participant's fixation locations in one block. Three distinct AOIs are clearly visible: left screen, iCub, right screen. All cluster outputs are available at https://osf.io/zxkwn. (Online version in colour.)

We used the binocular-individual threshold (BIT) algorithm [23] to classify fixations per individual per block. The BIT algorithm bases velocity thresholds on inter-individual and between-task variability in fixations and thus offers an objective method for eye-tracking data classification. Next, we intended to use the eyetracker's proprietary software to map the relevant areas of interest (AOIs) automatically, but it failed to classify the screens. Therefore, for each participant, the three relevant AOIs (left object, right object, iCub) were assigned by means of an offline *K*-means cluster analysis, using the SciPy Python library. This is a technique, often used in machine learning, that classifies each data-point (in our case fixation coordinates) based on the smallest distance to the gravitational centre of each group (in our case three AOIs). After each iteration, the algorithm then repositions the gravitational centre so that the sum of distances between each centre point and its data-points are minimal. This is repeated until repositions no longer change the classification and an optimum is reached (see figure 2 for an example *K*-means cluster output).

For each trial, we calculated the onset latency of return-to-face saccades as the time between the first fixation on the selected object and the subsequent first fixation on iCub's face. We discarded trials in which no fixation on one of the objects was detected, trials in which no gaze samples were detected prior to the fixations of interest, trials with anticipatory fixations on the object (i.e. before the cue), and trials with clear saccade undershoots or overshoots back on the robot's face. We also discarded trials in which object-fixations were too short. In other words, at times participants would look back at the robot before the robot completed its trajectory and the program was not able to register, and thus act on, the fixation back on the face. Trials with a delay of longer than 500 ms between the fixation back on the face and the trigger that prompted iCub to return its head to the centre were discarded accordingly.

We trimmed the means of the onset latencies of these return-to-face saccades to fall within 2 standard deviations of the mean per participant per condition (average 1.55% outliers, s.d. = 0.79). To preserve power, we excluded participant data if there were eight or fewer valid trials in the infrequent conditions; that is to say, the condition in which Jimmy did not follow the gaze and the condition in which Dylan did follow the gaze (removed *n* = 10, mean invalid trials = 65.6% across all blocks). Additionally, we discarded two more participants whose means were outliers (*z* > 2.5) in any of the four conditions. The values were now normally distributed; all Kolmogorov–Smirnov *p* values > 0.28, final *n* = 25; thus 12 participants were dropped in total.^2^ These data were subjected to a 2 (identity: Jimmy, Dylan)×2 (contingency: followed, unfollowed) ANOVA.

## 3. Results

### (a) Return-to-face saccades

**Figure 3.**
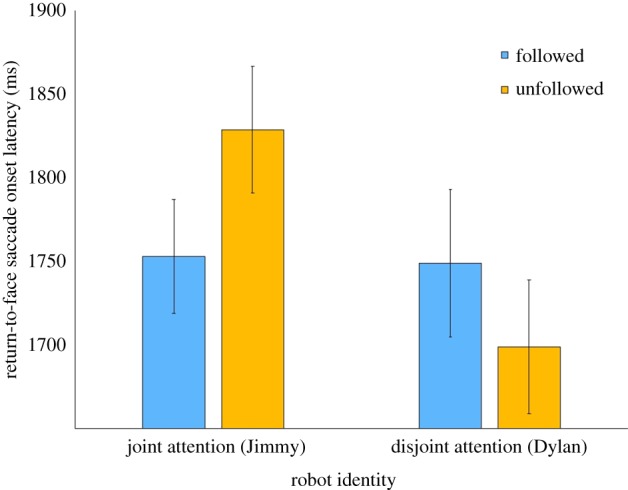
Mean onset latencies for the return-to-face saccades for each identity and contingency in milliseconds. Error bars: ±1 s.e.m. (Online version in colour.)

There were no main effects of identity (*p* = 0.37) or contingency (*p* = 0.52). However, there was an interaction effect between identity and contingency; *F*_1,24_ = 12.4, *p* = 0.002, *η*^2^ = 0.34 (figure 3). Follow-up paired-samples *t*-tests showed that for Jimmy (the robot with 80% of joint attention trials), the onset latencies of participants' return-to-face saccades were shorter (*M* = 1753 ms, s.d. = 401) when the robot followed than when it looked at the other object (*M* = 1829, s.d. = 444); *t*_24_ = 2.60, *p* = 0.016, *d* = 0.52. The other pairwise comparisons (respectively, Jimmy-followed—Dylan-followed and Dylan-followed—Dylan-unfollowed) were not significant; all *p* values > 0.055 (Dylan-followed *M* = 1749 ms, s.d. = 299; Dylan-unfollowed *M* = 1699, s.d. = 319).^3^

### (b) Questionnaires

#### (i) Intentional stance

Higher scores reflect more mentalistic as opposed to mechanistic explanations for the iCub's behaviour in the scenarios. Scores on the InStance questionnaire related to Jimmy (*M* = 40.4% mentalistic, s.d. = 18.1) did not differ significantly from the scores related to Dylan (*M* = 37.9% mentalistic, s.d. = 18.9); *p* = 0.40. Twelve participants rated Jimmy as more mentalistic and 13 participants Dylan. Adding these two fairly even groups as a between-subjects variable in our model showed there was no relationship between these scores and the return-to-face saccade onset-times.

#### (ii) Yoni

The 2 (identity: Jimmy, Dylan) × 2 (order: first-order theory of mind, second-order theory of mind) × 2 (theory of mind-type: affective, cognitive) repeated-measures ANOVA revealed a three-way interaction effect; *F*_1,24_ = 4.6, *p* = 0.04, $\eta_{p}^{2}=0.16$, but follow-up analyses did not yield further results. We found no other statistically significant differences for accuracy between the Yoni test relating to Jimmy and the Yoni test relating to Dylan; all *p* values > 0.07; all $\eta_{p}^{2}\;\mathrm{values}<0.14$.^4^

#### (iii) Godspeed

Participants did not anthropomorphize Jimmy more than Dylan (Jimmy: *M* = 16.7, s.d. = 3.6; Dylan: *M* = 16.2, s.d. = 4.1; *p* = 0.36), but attributed greater likeability towards the former, *t*_24_ = 2.7, *p* = 0.013, *d* = 0.54 (Jimmy: *M* = 20.5, s.d. = 3.5; Dylan: *M* = 18.7, s.d. = 3.8).^5^

#### (iv) Additional questions

Of the total of 25 participants whose data were analysed, 19 (76%; 78% for the entire sample) expressed a preference for Jimmy and 6 for Dylan. 21/25 participants indicated that they noted differences between the two identities related to choice [6], gaze-following [4], imitation [3] or other miscellaneous reasons [8]. Fifteen participants reported Jimmy to be the mentalistic robot and 10 the mechanistic.

## 4. Discussion

The interaction effect between identity and contingency for the return-to-face saccades indicates that attentional engagement with the robot was facilitated after initiating joint attention, but only if the robot typically followed the participant's gaze. This partly replicates previous findings that people are not merely sensitive to establishing *ad hoc* joint attention, but that this sensitivity depends on implicit expectations set by previous interaction [8,16,24,25]. Whereas a similar but screen-based study found a main effect in which return-to-face saccades were faster towards the joint-attention robot avatar than towards the disjoint-attention one [8], the current study suggests that an agent's joint-attention disposition drives further interaction. This implies that interaction with an embodied partner can evoke slightly different (social-)cognitive processes, relative to screen-based experimental paradigms, and we speculate that this interaction effect reported here is a direct effect of deeper social engagement with embodied robots compared with two-dimensional avatars. In other words, our participants might have been more engaged in the interaction, and thus more sensitive to when the robot followed their gaze or did not. This sensitivity to online behaviour was strengthened when they formed particular expectations of positive social interaction with the typically following robot. This is analogous with studies that found similar results in human–robot interaction compared with screen studies [26] and also with studies that found different gaze behaviour in real-life settings with human presence relative to humans on a screen [11,15,27] and extends the invitation to carefully examine if social cognition in a realistic scenario follows the same principles that were reported in perhaps less ecologically valid experiments (e.g. [15,28]). Furthermore, our findings provide evidence that re-establishing eye-contact after episodes of joint attention (closing the loop) is facilitated when these episodes occur frequently as people appear to be sensitive towards this frequency.

Secondly, we hypothesized that Jimmy, the joint-attention identity, would evoke more favourable subjective attributions than Dylan, the disjoint-identity robot. Seeing that participants gave significantly higher likeability ratings and indicated a greater preference for ‘Jimmy’ than for ‘Dylan’, we confirm this hypothesis. However, participants did not anthropomorphize one identity more than the other. This suggests that contingent gaze behaviour in robots is perhaps too subtle to be perceived as more human-like, and other factors such as appearance and movement kinematics might play a bigger role required for anthropomorphism [29]. But, one could argue that likeability and preference are typically human-like attributions and in that respect we replicate findings that gaze behaviour is a strong predictor of positive personal attributions [5,7,8].

Whereas personal attributions were generally more favourable towards Jimmy, the joint-attention identity, we found no such differences in adopting the intentional stance, which can be explained in three ways. Firstly, in the literature theory of mind is typically described as a characteristic trait [30]. In other words, individuals have a high or low de facto ability to attribute mind to other agents regardless of subtle behavioural differences between the agents. Even if the degree of adopting the intentional stance can vary as a function of identity [17], the difference in attributed ‘identities’ may have been too implicit between the two robots, which only differed on gaze-contingency but which were highly similar otherwise. Secondly, perhaps the photographical (InStance questionnaire) and schematic (Yoni test) representations of the robot were semantically and temporally too distinct from the embodied agents that the participants interacted with, to detect any such differences in adopting the intentional stance. Finally, it is worth noting that at the time of the experiment, the Intentional Stance questionnaire was still in development. This questionnaire may prove to be a useful tool in detecting how readily individuals adopt the intentional stance towards iCub. However, how this adoption can be manipulated is still to be examined.

Potential limitations of our study include the fact that ‘changing the robot identity’ may not be high in believability. We attempted to increase this by referring to the identities by their names from the beginning of the experimental sessions, by trying to give the impression that the experimenter was working hard to achieve this change while the participants were doing their first round of questionnaires, and by having the robots introduce themselves with their voice before each block. Perhaps a future study could use two actual different robots in a counterbalanced design, even though this will bring forward other practical challenges and may introduce new confound variables.

Finally, our study is a proof-of-concept of the idea that traditional paradigms of experimental cognitive psychology can be implemented in a more naturalistic human–robot interaction set-up, without compromising experimental control. Although this work has been the first to our knowledge to meet the challenge of simultaneously integrating an online eyetracker with stimulus presentation software as well as with an embodied robot (which exhibited eye movement behaviour contingent on the eye movements of the participants), we managed to successfully meet the challenge in experimental design/control and data collection. Furthermore, we also overcame the challenges involved in the off-line data processing. Most notably, as the proprietary automatic mapping of AOIs did not perform as desired, we came up with the solution of using a machine learning technique to detect the three AOIs in each participant's gaze data. Naturally this was not as fine-grained as static, pixel-defined AOIs typically used in desktop eyetracking, but from checking against the eyetracker recordings, *K*-means clustering proved to be an efficient and accurate method. Furthermore, there was a spatial and temporal heterogeneity in eye movements between participants. This made it difficult to specify an overarching catch-all fixation filter with the ideal signal-to-noise ratio, which is why we opted for individual thresholds with the BIT algorithm. Notwithstanding the potential noise in the data however, to the best of our knowledge this is the first time that a gaze-contingent paradigm has been successfully implemented with mobile eyetracking and an embodied robot platform. We therefore provided evidence that the sampling rate and spatial resolution of the latest mobile eyetrackers are adequate for studying the subtle attentional mechanisms previously thought quantifiable only with stringent screen-based paradigms.

In conclusion, our results show that attention towards those with whom we typically establish joint attention is facilitated when our gaze is followed, and that we have a preference towards those agents. However, it is not yet clear whether we are more likely to adopt intentional stance towards those who display more contingent joint attention than those who do not. Furthermore, we demonstrated the feasibility of mobile eyetracking as a tool to carry out advanced gaze behaviour studies in more naturalistic settings which use embodied robots as ‘stimuli’, thereby forming a viable opportunity to increase ecological validity while maintaining excellent experimental control within the walls of the laboratory.

## Footnotes

1 As a result, the experimenter was not blind towards the different robot identities. The experimenter acted neutrally and the same during both introductions, however.

2 As this may appear a high exclusion rate, we report here that when we employed a less stringent, yet perhaps more subjective inclusion criterion resulting in a final *N* of 32 participants, we found the same effects with similar effect sizes.

3 These effects were replicated when we entered median return-to-face saccade onset-times into these models.

4 There was a main effect of order; *F*_1,24_ = 14.2, *p* = 0.001, $\eta_{p}^{2}=0.37$. Participants made fewer errors on trials with first-order theory of mind scenarios (accuracy *M* = 0.94, s.e. = 0.027) than with second-order ToM scenarios (*M* = 0.83, s.e. = 0.026). A 2 (identity: Jimmy, Dylan) × 2 (eyes: leading, misleading) ANOVA yielded no significant results either, indicating that sensitivity to the gaze-direction of the Yoni-avatars was not affected by the interaction with the iCub identities.

5 Similar statistics were found when we conducted the Intentional Stance, Yoni and Godspeed analyses on the entire sample of 37 participants.

## Data accessibility

The scripts and data used and reported in this study can be accessed online via https://osf.io/zxkwn.
